# Photoreceptive retinal ganglion cells control the information rate of the optic nerve

**DOI:** 10.1073/pnas.1810701115

**Published:** 2018-11-28

**Authors:** Nina Milosavljevic, Riccardo Storchi, Cyril G. Eleftheriou, Andrea Colins, Rasmus S. Petersen, Robert J. Lucas

**Affiliations:** ^a^Faculty of Biology, Medicine and Health, University of Manchester, Manchester M13 9PT, United Kingdom;; ^b^Center for Synaptic Neuroscience and Technology, Istituto Italiano di Tecnologia, 16132 Genova, Italy

**Keywords:** neural coding, information, melanopsin, ipRGC, retina

## Abstract

Information transfer in the brain relies upon energetically expensive spiking activity of neurons. Rates of information flow should therefore be carefully optimized, but mechanisms to control this parameter are poorly understood. We address this deficit in the visual system, where ambient light (irradiance) is predictive of the amount of information reaching the eye and ask whether a neural measure of irradiance can therefore be used to proactively control information flow along the optic nerve. We first show that firing rates for the retina’s output neurons [retinal ganglion cells (RGCs)] scale with irradiance and are positively correlated with rates of information and the gain of visual responses. Irradiance modulates firing in the absence of any other visual signal confirming that this is a genuine response to changing ambient light. Irradiance-driven changes in firing are observed across the population of RGCs (including in both ON and OFF units) but are disrupted in mice lacking melanopsin [the photopigment of irradiance-coding intrinsically photosensitive RGCs (ipRGCs)] and can be induced under steady light exposure by chemogenetic activation of ipRGCs. Artificially elevating firing by chemogenetic excitation of ipRGCs is sufficient to increase information flow by increasing the gain of visual responses, indicating that enhanced firing is a cause of increased information transfer at higher irradiance. Our results establish a retinal circuitry driving changes in RGC firing as an active response to alterations in ambient light to adjust the amount of visual information transmitted to the brain.

## Significance

Noise in the visual signal falls as ambient light increases, allowing the retina to extract more information from the scene. We show here that a measure of ambient light produced by the small number of inner retinal photoreceptors [intrinsically photosensitive retinal ganglion cells (ipRGCs)] regulates intrinsic rates of spike firing across the population of retinal ganglion cells that form the optic nerve. Increased firing at higher irradiance allows the ganglion cells to convey more information. Our findings reveal a potential mechanism for increasing visual performance at high ambient light and show that changes in maintained activity can be used to provide proactive control over rates of information flow in the CNS.

As information in the nervous systems is conveyed by action potentials (spikes) it should come as no surprise that rate of spike firing and information transfer are closely correlated (1–3). That relationship is often thought of as being reactive—the process of conveying more information imposes higher firing. However, an alternative possibility is that the excitability of neural systems may be proactively adjusted to allow enhanced information transfer under conditions in which this would be advantageous, while saving energy in conditions when high information is not strictly required.

**Fig. 1.**
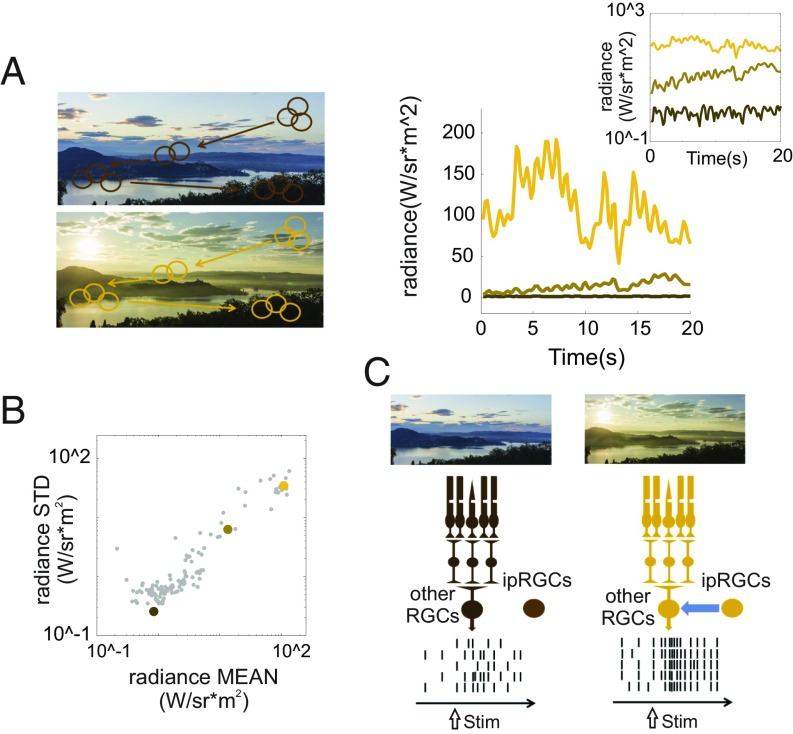
A model for enhanced visual information transfer at higher ambient light. (*A*) During visual exploration, receptive fields of RGCs move across visual patterns [shown to *Left* as simulated shifts in the location of a receptive field (circle) across the scene over time]. The amplitude of resultant variations in radiance is positively correlated with ambient light (shown to *Right*, and *Inset* as semilogarithmic plot, for three representative recordings of radiance during active viewing of the same natural scene at different times of day; brown, tan, and yellow lines for dim, moderate, and bright ambient light, respectively). (*B*) This fundamental correlation between ambient light (mean radiance) and its variability over space and time (SD of radiance) is apparent in a larger population of radiance recordings across different times of day (*n* = 126 independent recording epochs, each lasting 20 s; colored dots correspond to the data shown in the panel). As photon noise scales as the square root of mean photon flux, this corresponds to an increase in signal/noise for the visual input and therefore in the amount of visual information available to encode. (*C*) Our working hypothesis is that the measure of ambient light provided by ipRGCs is used as a signal to scale the tonic firing rate of RGCs and that this has the effect of increasing the number of spikes available to convey visual information at high irradiance [apparent as increased temporal structure in simulated raster plots (at *Bottom*) for repeats of the same visual stimulus (arrow)].

One element of the nervous system in which such proactive control of neural activity is potentially functionally relevant and physiologically plausible is the inner retina. In terms of function, the amount of visual information available for the retina to encode is positively correlated with ambient light intensity (Fig. 1 *A* and *B*). Consequently, a mechanism to adjust retinal activity according to ambient light could serve the purpose of optimizing information transfer to a predictable change in functional demands. In terms of physiology, the retina contains a particular class of neuron [intrinsically photoreceptive retinal ganglion cells (ipRGCs)] that is specifically optimized for encoding ambient light (4–6). While many retinal ganglion cells (RGCs) lack the spatiotemporal averaging capacity required to track changes in background light (7), ipRGCs express sustained responses to steady levels of illumination (6, 8) and have previously been shown to influence maintained activity in the visual thalamus according to irradiance (9). Moreover, ipRGCs have several routes by which they can influence the wider retinal circuitry (10–13), leaving them well placed to provide irradiance-dependent control over retinal activity. Here, we therefore set out to ask whether ipRGCs adjust the intrinsic firing rate of the retinal output neurons (RGCs) according to ambient light levels as a mechanism of proactively controlling information transfer capacity of the visual system (Fig. 1*C*).

We find that gradual increments in ambient light (irradiance ramps) are indeed accompanied by increases in RGC firing and that this is associated with an increase in information rate. The change in firing occurs in absence of any other visual stimulus and is partially disrupted in the absence of melanopsin (the photopigment of ipRGCs), indicating that it is a genuine change in intrinsic activity as a response to alterations in ambient light, and at least partly attributable to ipRGCs. Chemogenetic activation of ipRGCs recapitulates increases in both firing and information rates. This latter finding not only confirms the importance of ipRGCs in setting RGC firing, but establishes a causative relationship between firing and information rates by showing that increasing firing is sufficient to enhance information in the absence of any change in the visual environment. These data reveal a mechanism for increasing visual information at higher ambient light and establish the potential for proactive control of neuronal firing to be used to scale information flow in the nervous system according to predictable changes in demand.

## Results

### Firing Rates in the Ganglion Cell Layer Are Defined by Irradiance.

**Fig. 2.**
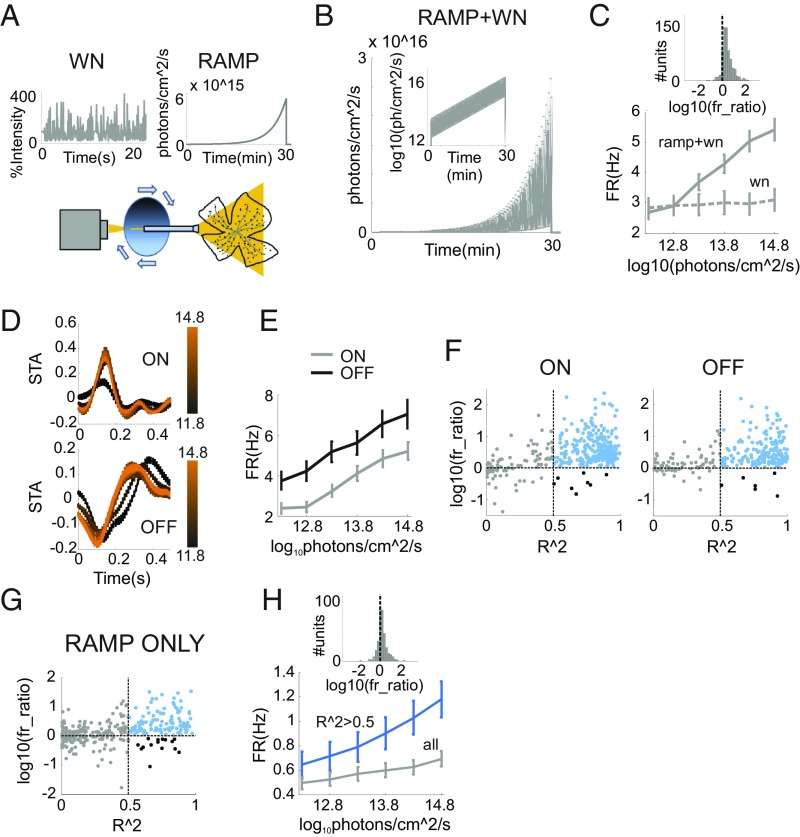
Increase in firing rates across RGC population correlates with irradiance. (*A*) Schematic of our ramp-plus-white noise (RAMP+WN) visual stimulation produced by a light source generating fast radiance fluctuations (WN) and passed through a slowly moving neutral-density filter wheel to produce a gradual change in irradiance (RAMP). These two stimulus components recapitulate the radiance SD and the radiance MEAN signals in Fig. 1*C*, respectively. (*B*) Irradiance values as function of time during the RAMP+WN stimulus (as semilog plot in *Inset*). (*C*) Time-averaged firing rate (mean ± SEM) as function of irradiance (0.5 log-units of irradiance per epoch) across the population of RGCs exposed to the RAMP+WN stimulus (solid line; *n* = 790), or control condition in which irradiance held constant (WN; dashed line; *n* = 309). Distribution of logarithmic transformed firing-rate ratios between the start and end of the ramp (log_10_fr_ratio_; *Inset*) is unimodal, and most units express an increase in firing [log_10_(fr_ratio_) > 0]. (*D*) STAs (mean ± SEM) for the white-noise stimulus in units with ON (*Top*; *n* = 406) or OFF (*Bottom*; *n* = 303) response polarity, across six epochs of the irradiance ramp (color code for lines at *Left* in log photons per square centimeter per second). (*E*) As in *C*, plotting ON and OFF units separately (gray and black lines, respectively). (*F*) The relationship between irradiance and time-averaged firing was captured for individual units as log_10_(fr_ratio_) and as the *R*^2^ of the log:linear relationship between firing rate and irradiance (*Methods*). Scatter plots for these parameters for ON and OFF units revealed that many more units with high *R*^2^ (>0.5) had increases (blue dots) than decreases (black dots) in firing rate (all units with *R*^2^ < 0.5 shown in gray). (*G* and *H*) As in *F* and *C*, but for RGCs presented with the RAMP stimulus without superimposed WN for all units and units with *R*^2^ > 0 (*n* = 455 and 124; gray and blue lines, respectively).

We used a multielectrode array (MEA) to make extracellular recordings across the ganglion cell layer (GCL) of mouse retinal explants exposed to a repeated sequence of temporal white noise superimposed upon a gradual, high-amplitude, irradiance ramp (irradiance range, 11.8–14.8 log_10_ photons⋅cm^−2^⋅s^−1^; duration, 30 min; Fig. 2*A*). This protocol, was designed to simultaneously elicit responses to slow changes in ambient light spanning low- to mid-photopic range [simulated by the ramp and based on dawn recordings (9)] and higher-frequency visual signals (simulated by the white-noise stimulus). The amplitude of the white-noise stimulus scaled with irradiance (Fig. 2*B*) to retain the fundamental relationship between the mean and SD of light intensity observed in the real world (Fig. 1*B*).

To assess the impact of changing irradiance on maintained activity, we first computed the mean firing rate across the ramp for 790 light-responsive single units isolated from six retinas. This revealed an increase in mean firing as a function of irradiance (Fig. 2*C*; *P* ∼ 0, df = 5, χ^2^ = 95.9, *n* = 790; *P* = 0.6, df = 5, χ^2^ = 3.63, *n* = 309; Kruskal–Wallis test for RAMP+WN and control for WN only, respectively). The distribution of ratios between firing rates at beginning and end of the ramp (*Methods*) was unimodal (Fig. 2*C*, *Inset*; *P* = 0.975; dip = 0.00976; *n* = 790; 10,000 bootstrap samples; Hartings dip test for bimodality), indicating that the ramp-driven increase in firing rate was widespread across the whole population, rather than specific for a subset of neurons. However, as this effect was also clearly more apparent in some neurons than others, we asked whether the impact of irradiance was different across a major division in RGC function—between neurons with ON vs. OFF responses to radiance increments. We computed spike-triggered averages (STAs) for the white-noise stimulus to identify ON and OFF units. Of 709 units with statistically significant STAs, 406 had ON- and 303 OFF-type responses (Fig. 2*D*). Both ON and OFF populations showed progressive increases in mean firing rate across the ramp (Fig. 2*E*; ON: *P* ∼ 0, df = 5, χ^2^ = 81.6, *n* = 406; OFF: *P* ∼ 0, df = 5, χ^2^ = 40.19, *n* = 303; Kruskal–Wallis test).

To determine the extent to which individual units displayed a systematic change in firing rate across the ramp, we next calculated goodness of fits (*R*^2^) for a log:linear relation between firing rate and irradiance for single units. High *R*^2^ values indicate progressive changes in firing, and low values, either minimal or more discontinuous/stochastic variations in activity. Units with high *R*^2^ values were strongly biased toward increases in firing across the ramp (Fig. 2*F*; ON: *P* ∼ 0, zval = 16.25, *n* = 297; OFF: *P* ∼ 0, zval = 13.67, *n* = 212; sign test for change in firing in either ON or OFF units with *R*^2^ > 0.5). Indeed, of 406 ON units in these recordings, 297 had *R*^2^ > 0.5 and 289 of these increased firing; while of 303 OFF units, 212 had *R*^2^ > 0.5 and 206 of those increased firing.

To determine whether the increase in firing across the ramp was a genuine change in intrinsic activity induced by the alteration in ambient light [as opposed to a consequence of enhanced responses to the superimposed white noise whose amplitude also scaled with irradiance (Fig. 1*B*, radiance SD)], we presented a featureless irradiance ramp, without superimposed white noise (duration, 13 min; *n* = 455 units from seven retinas). We found that a significant fraction of units expressed a log-linear relation across this ramp (Fig. 2*G*; 31%, *n* = 142/455). The majority of those units increased their activity and only a few decreased (27% vs. 4% with *R*^2^ > 0.5; *P* ∼ 0; zval = 8.8; sign test for difference in the number of units with increase vs. decrease in firing rates). As previously observed for the ramp-plus-white noise data, we found no clear bimodality in the distribution of firing rate changes, indicating that irradiance responses were not restricted to a particular subset of RGCs (Fig. 2*H*, *Inset*; *P* = 0.537; dip = 0.019; *n* = 455; 10,000 bootstrap samples; Hartings dip test for bimodality). A comparison with the ramp-plus-white noise dataset indicated that ganglion cell firing rates were impacted by both the white noise and the irradiance ramp. Thus, while firing rates increased across the simple ramp, they were overall lower than under the ramp-plus-white noise stimulus (Fig. 2 *C* and *H*). The fraction of units showing a progressive increase in firing was also smaller in the absence of white noise (27% vs. 64%), but the relative increase in firing for units with *R*^2^ > 0.5 for the log:linear model was comparable (2.08- and 2.15-fold increase for ramp and ramp-plus-white noise, respectively).

### ipRGCs Control RGC Firing Rates.

**Fig. 3.**
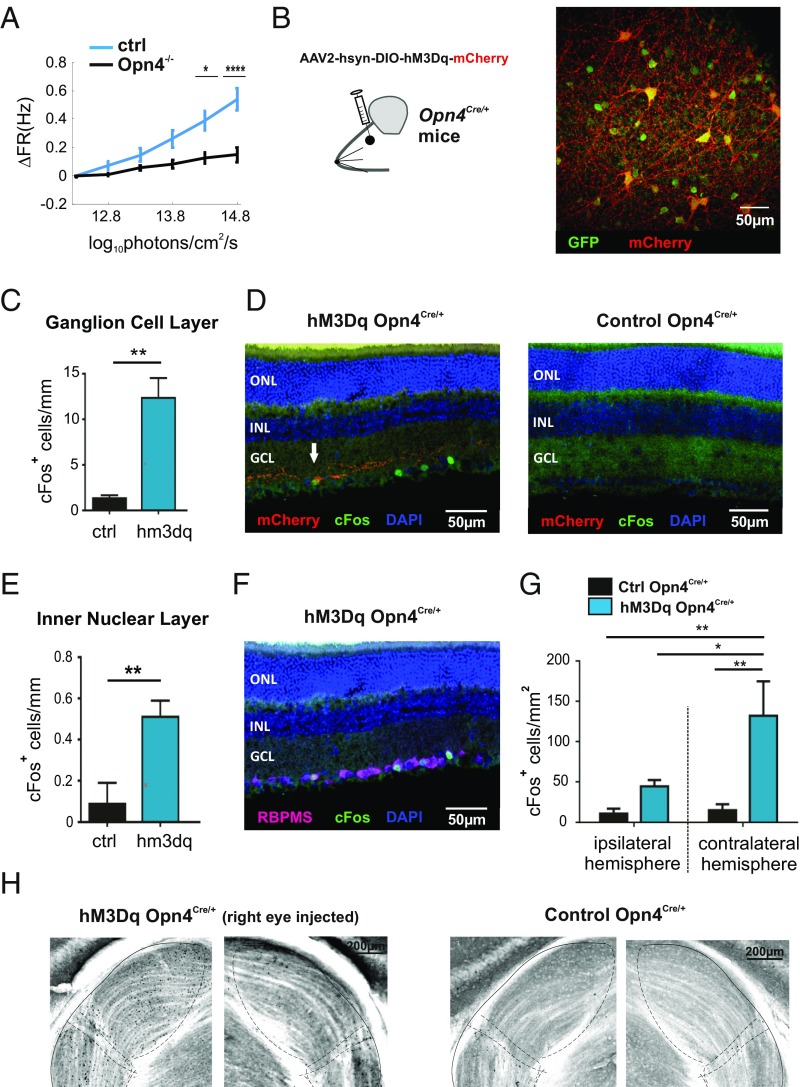
ipRGCs contribute to modulations in RGC-maintained activity. (*A*) Changes in firing rate induced by a gradual irradiance ramp (11–14.8 log photons⋅cm^−2^⋅s^−1^ over 13 min) were substantially deficient in *Opn4*^*−/−*^ mice (black lines) compared with controls (blue lines). Statistically significant differences were determined after Bonferroni correction (*P* = 0.18, 0.25, 0.02, 0.004, and 3 × 10^−9^; zval = 1.33, 1,15, 2.28, 2.88, and 5.89, rank sum tests; *n* = 455 and 662, control and *Opn4*^*−/−*^ mice, respectively). (*B*) Intravitreal injections of viral vector (AAV2-hsyn-DIO-hM3Dq-mCherry) in *Opn4*^*Cre/+*^*;Z/EGFP* mice (*Inset*) resulted in immunoreactivity for mCherry (red) in a subset of GFP-positive ipRGCs (green). Shown here for representative retinal whole mount. (Scale bar: 50 μm.) (*C*) Mean (±SEM) c-Fos–positive cells per millimeter length of retinal section after CNO administration (5 mg/kg, i.p.) was higher in hM3Dq-expressing (blue bar; *n* = 6) than control (black bar; *n* = 4) retinas in ganglion cell layer (GCL) and inner nuclear layer (INL). Unpaired *t* test; **P < 0.01. (*D*) Immunohistochemical staining of representative retinal sections showing c-Fos (green) expression in hM3Dq-expressing (*Left*) but not control (*Right*) retina held in dark but exposed to CNO extending beyond hM3Dq-expressing (mCherry immunoreactive, red) ipRGCs (arrow). (*E*) Same as *C* for INL. (*F*) Representative retinal section from a hM3Dq-expressing mouse following CNO administration stained for c-Fos (green) and RBPMS (purple) immunoreactivity showing all c-Fos–positive cells also positive for the ganglion cell marker RBPMS. Blue shows DAPI nuclear counterstain. (*G*) Mean (±SEM) c-Fos–positive cells per square millimeter in ipsilateral and contralateral dLGN after CNO administration (5 mg/kg, i.p.) in hM3Dq-expressing (blue bars; *n* = 6) and control (black bars; *n* = 6) mice (two-way ANOVA, *P < 0.05 and **P < 0.01). (*H*) Representative micrographs of coronal sections through lateral geniculate nucleus (approximate margins for dorsal and ventral subdivisions and intergeniculate leaflet shown with dotted lines) contralateral (*Left*) or ipsilateral (*Right*) to the eye injected with hM3Dq virus (*Left*) or vehicle control (*Right*) show c-Fos immunoreactivity (dark) in the former but not the latter following CNO administration. *P < 0.05, ***P < 0.0001.

As a first test of the hypothesis that ipRGCs contribute to irradiance-dependent changes in GCL activity, we asked whether this behavior was disrupted in melanopsin-knockout mice (*Opn4*^*−/−*^). Because ipRGCs integrate information from rods and cones with their own melanopsin-dependent intrinsic light response, irradiance responses are typically disrupted rather than abolished following melanopsin loss (9, 14, 15). This turned out also to be the case for the change in GCL firing. As firing activity in dimmest condition was different between these strains (0.35 ± 0.04 SEM, 0.49 ± 0.05 SEM; *P* ∼ 0, zval = 6.442; *n* = 662 and 455, rank sum test; *Opn4*^*−/−*^ and control animals), we compared the change in firing rate across the ramp. While the irradiance ramp did elicit an increase in firing in *Opn4*^*−/−*^ retinas, the magnitude of this effect was significantly reduced compared with that of melanopsin-sufficient controls (Fig. 3*A*).

As a next test of ipRGC involvement in setting RGC firing, we set out to determine the impact of light-independent, chemogenetic activation of ipRGCs (16–18) on GCL activity. We injected a recombinant AAV2 vector [AAV2-hSyn-DIO-hM3D(Gq)-mCherry] driving cre-dependent expression of the hM3Dq receptor and an mCherry reporter into the vitreal space of *Opn4*^*Cre/+*^ mice (Fig. 2*B*, *Inset*). This resulted in expression of hM3Dq in around 35% of ipRGCs (Fig. 3*B*). This level of expression is in accordance with our previous experience (16, 17) in which the projection targets of infected ipRGCs suggested no strong bias for any particular ipRGCs subtype. To determine whether activation of this fraction of ipRGCs altered GCL activity (in the absence of any visual stimulus), we first used c-Fos excitation mapping. The hM3Dq agonist clozapine N-oxide (CNO) (19) was applied (5 mg/kg, i.p., injection) to mice held in the dark overnight and throughout the experiment. Ninety minutes after injection, animals were perfused, and retinas and brains were collected for immunocytochemical identification of the c-Fos marker of neuronal activation. Given evidence that CNO is not entirely biologically inert at the concentrations required for CNS activation (20, 21), we included a cohort of control *OPN4*^*Cre/+*^ mice receiving intravitreal injection with virus-free vehicle.

Retinal sections from hM3Dq and control *OPN4*^*Cre/+*^ mice were stained for c-Fos (Fig. 3*D* and *SI Appendix*, Fig. S1 *A* and *B*). There were significantly more c-Fos–positive nuclei in both the GCL and inner nuclear layer (INL) of hM3Dq-expressing retinas (Fig. 3 *C* and *E*; two-tailed unpaired *t* tests, *P* = 0.0028 and *P* = 0.0071 for GCL and INL, respectively). All mCherry-positive (hM3q-expressing) cells were also c-Fos–positive, but c-Fos was not restricted to these transduced ipRGCs, as for every mCherry-positive cell there were approximately eight mCherry-negative c-Fos–positive cells across INL and the GCL. These findings indicate that chemogenetically activated ipRGCs in turn excite many other cells in the GCL and INL (*SI Appendix*, Fig. S1*C*). In mice, both amacrine and ganglion cells can appear in both of these layers. To determine the degree to which the c-Fos signal appeared in ganglion cells, we counterstained with an RGC marker [RNA-binding protein with multiple splicing (RBPMS) (22)]. This revealed that the vast majority (at least 90%) of c-Fos–positive cells were indeed ganglion cells (Fig. 3*F* and *SI Appendix*, Fig. S1 *A* and *B*).

Enhanced firing of RGCs should be reflected in activation of retinorecipient targets in the brain. As an additional confirmation of RGC activation in our chemogenetically treated animals, we checked for c-Fos expression in the major thalamic target for retinal innervation, the dorsal lateral geniculate nucleus (dLGN), which receives input from a diverse population of RGCs (23). We found increased numbers of c-Fos–positive nuclei in dLGN of hM3Dq-expressing, compared with control, *OPN4*^*Cre/+*^ mice, both ipsilateral and (especially) contralateral to the injected eye (Fig. 3 *G* and *H*; two-way ANOVA with Dunnett correction for multiple comparisons; *n* = 6). These data are consistent with our recent finding of irradiance-dependent increases in firing rate of the mouse dLGN (9).

**Fig. 4.**
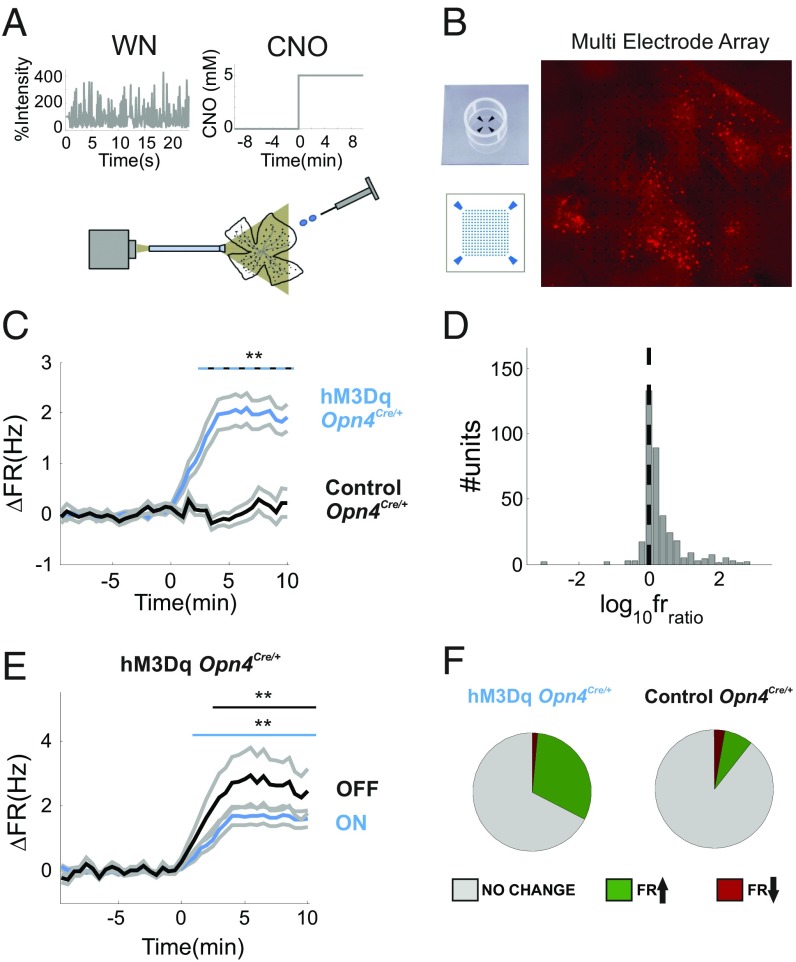
Chemogenetic activation of ipRGCs enhances maintained firing across the RGC population. (*A*) Schematic of our visual-chemogenetic stimulus. A white-noise (WN) stimulus was presented at constant mean irradiance (11.5 log_10_ photons⋅cm^−2^⋅s^−1^) with timed CNO delivery. (*B*) Representative image for a whole-mount retina rec orded under our MEA system. mCherry fluorescence spanning large regions of the recording array could be observed. (*C*) Mean ± SEM change in time-averaged firing rate (ΔFR over mean before CNO administration) for hM3Dq-expressing (blue line) and control (black line) retinas following CNO application (5 µM) commencing at time 0. The top blue-black dashed line indicates significant differences in ΔFR (**P < 0.01, rank sum tests, Bonferroni’s correction). (*D*) The distribution of logarithmic ratios between firing rates (log_10_fr_ratio_) from hM3Dq-expressing retinas, before and after CNO delivery, is unimodal, and most units express an increase in firing rate (log_10_fr_ratio_ > 0). (*E*) As in *B*, plotting ON and OFF units from hM3Dq retinas (lines above indicate significant increases in firing; *P* < 0.01, sign rank tests, Bonferroni’s correction). (*F*) Pie charts presenting percentage of units that showed increase, decrease, or no change in firing rate upon CNO application in hM3Dq (*Left*) and control (*Right*) retinas.

The c-Fos experiments thus suggest that chemogenetic activation of ipRGCs enhances activity in a substantial fraction of ganglion cells. For a more direct assessment of the impact of this manipulation on firing rates, we performed MEA recordings from the GCL of retinal explants from hM3Dq-expressing and vehicle-injected mice. Throughout recordings, retinas were exposed to a repeated sequence of full-field white noise (Fig. 4 *A* and *B*) at an irradiance (mean, 11.5 × log_10_ photons⋅cm^−2^⋅s^−1^) at which melanopsin activation should be slight (5). After 40 min of recordings under perfusion with standard artificial cerebrospinal fluid (aCSF), we added 5 µM CNO. In total, we recorded from nine hM3Dq-expressing and six control retinas (hM3Dq: from eight and five mice, respectively). CNO administration was associated with a significant increase in time-averaged firing rate in hM3Dq, but not control, retinas [Fig. 4*C*; hM3Dq: *P* ∼ 0, zval = 6, *n* = 393 units from nine retinas (eight mice); controls: *P* = 0.41, zval = 0.873, *n* = 309 units from six control retinas (five mice); rank sum test comparing unit’s firing rate over 10 min before vs. 10 min after CNO application]. There was no clear multimodality to the distribution in firing-rate ratios before compared with after CNO (Fig. 4*D*; *P* = 0.998; dip = 0.01; *n* = 393; 10,000 bootstrap samples; Hartings dip test for bimodality). In this way, the impact of chemogenetic activation of ipRGCs did not appear to be restricted to a clearly defined subpopulation of RGCs but, in common with the response to an irradiance ramp, was expressed across the ganglion cell population. As with irradiance-dependent changes in firing, this CNO response was apparent in units with both ON and OFF response polarity (Fig. 4*E*).

To determine what fraction of units displayed a significant increase in firing, we compared mean firing rates over 40 repeats of the white-noise stimulus on either side of CNO administration for each single unit. Thirty-one percent of units from hM3Dq retinas (122/393 units) showed a statistically significant increase in firing post-CNO, with only 1.5% (6/393 units) showing a significant reduction over this time frame (Fig. 4*F*; rank sum test with Bonferroni-corrected α = 0.05). In the control retinas, the figures were 7.7% and 3% (24/309 and 9/309 units, respectively; Fig. 4*F*). Furthermore, a comparison of firing-rate profiles for the 20% units with highest increase in activity following CNO in the two groups revealed a substantially larger increase in the hM3Dq population (*SI Appendix*, Fig. S2*A*). Thus, not only were increases in firing more common in hM3Dq retinas, but, when present, their amplitude was qualitatively different.

### Increases in Firing Rate Enhance Information Rate.

**Fig. 5.**
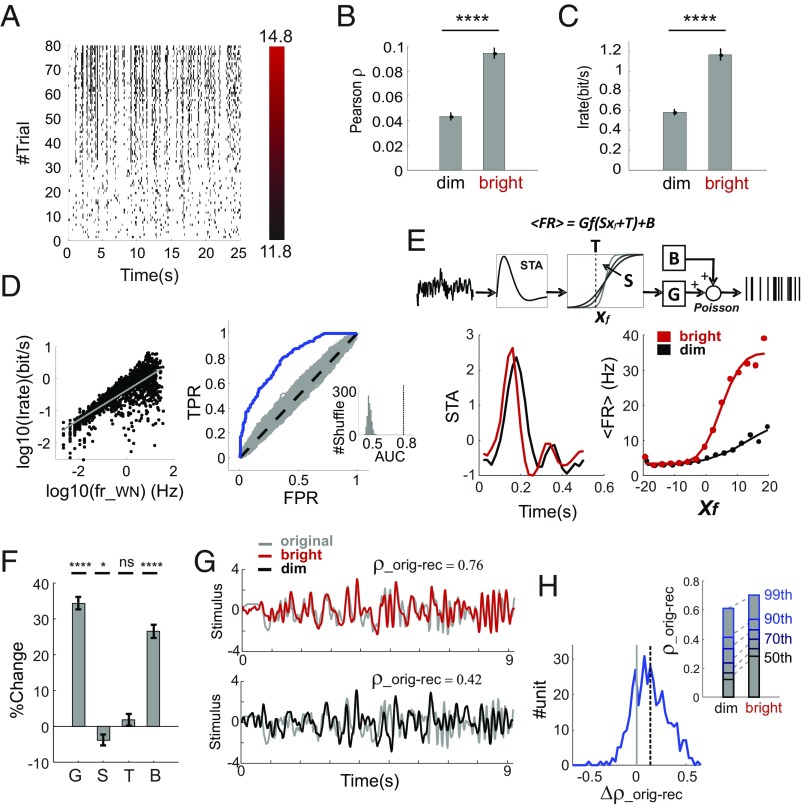
Impact of irradiance-driven increases in firing on neural coding. (*A*) Binarized trial bin counts for a representative unit from the RAMP+WN experiment to repeated presentations of the WN sequence. (*B*) Trial-to-trial reproducibility for units expressing increase in firing rates across the ramp were significantly higher in the last 40 trials (bright: 13.3–14.8 log_10_ photons⋅cm^−2^⋅s^−1^) compared with the initial 40 trials (dim: 11.8–13.3 log_10_ photons⋅cm^−2^⋅s^−1^). (*C*) As in *B*, but for information rate. (*D*) Firing and information rates are positively correlated (*Left*), and changes in firing rates across the ramp are predictive of changes in information rate (*Right*; *Inset* represents the shuffle test). (*E*) Schematic representation for the LNP model (*Top*) used to separately fit stimulus–response relations in dim- and bright-light conditions. STA (*Bottom Left*) and static nonlinearity (*Bottom Right*) in dim and bright conditions for a representative neuron. (*F*) Increases in response gain and baseline (G and B, respectively) were observed at high irradiance, while sensitivity and threshold (S and T, respectively) did not change (*P* ∼ 0 for G and B, *P* = 0.038 and 0.941 for S and T; *n* = 790; sign test). (*G*) Application of the model to reconstruct a stimulus based on firing patterns under bright (*Top*) and dim (*Bottom*) conditions, in the representative neuron [from *E*; colored and gray lines, reconstructed and original stimulus, respectively; correlation coefficients between reconstructed and original (ρ_orig-red_) shown above]. (*H*) Histogram of differences in ρ_orig-red_ at high vs. low irradiance (Δρ_orig-red_) in neurons showing increases in firing across the ramp, revealing a significant increase in ρ_orig-red_ at high irradiance (*P* ∼ 0; *n* = 385; sign test; dashed line shows mean). *Inset* shows quantiles for the distribution of ρ_orig-red_ for dim and bright condition. *P < 0.05, ****P < 0.0001.

To determine the impact of increases in ganglion cell firing on visually evoked activity, we turned first to recordings under the ramp-plus-white noise stimulus. An analysis of trial-to-trial correlations for repeated presentations of the white noise revealed a significant increase in response reproducibility at higher irradiances (Fig. 5 *A* and *B*; *P* ∼ 0; zval = 9.73; *n* = 790; rank sum test). The joint increase in firing rate (Fig. 2*C*) and reproducibility across the ramp in this dataset implies an increase in visual information. We quantified this effect in terms of Shannon’s mutual information (*Methods*). We found that information rates (bits per second) were on average higher at higher irradiance (*P* ∼ 0; zval = 6.39; *n* = 790; rank sum test). According to our hypothesis, this increase in information should be closely tied to changes in firing rate, and this was indeed the case. Thus, the information increase was apparent in units whose firing rate rose across the irradiance ramp (Fig. 5*C*; *P* = *P* ∼ 0, zval = 8.6, *n* = 425, rank sum test for units with log:linear increase in firing) but not in the remainder of the population (*SI Appendix*, Fig. S2*B*; *P* = 0.27, zval = 1.09, *n* = 326, rank sum test for remaining units). Moreover, there was a clear correlation between information and firing rates at single-unit level (Fig. 5*D*, *Left*; Spearman’s ρ = 0.62, *P* ∼ 0). Finally, changes in information for single units across the ramp could be predicted from alterations in their firing rate [Fig. 5*D*, *Right*; area under curve (AUC) = 0.79, *P* ∼ 0, shuffle test].

If additional spikes at higher irradiance were simply explained by an augmented baseline activity, then we would expect information to decrease. To explain the increase in information that we actually observed, we asked whether the gain of visual responses also improved [as previously reported in the dLGN (9)]. To estimate response gain, we fitted the activity of individual neurons with a linear-nonlinear–Poisson (LNP) model (24) (Fig. 5*E*, *Top*; *Methods*), in which the visual stimulus was filtered by a description of the feature preference for that neuron (the STA), transformed by a nonlinear function [parameterized by sensitivity (S) and threshold (T)], and multiplied by response gain (G) to produce a probability of firing. Stimulus-unrelated changes in baseline firing were accounted for by a final additive parameter (B). Application of this model to a representative neuron is summarized in Fig. 5*E* (*Bottom*), in which STAs at high and low irradiance appear to *Left*, and model fits to observed mean firing rate as a function of the filtered stimulus are shown to *Right*. Across the population of neurons, there were significant increases in both response gain and baseline firing across the ramp (Fig. 5*F*; *P* ∼ 0 for both; *n* = 423; sign tests), indicating that indeed effects of irradiance extended beyond augmentations in baseline firing to potentiate stimulus-evoked activity. Other parameters of the static nonlinearity (S and T) showed little or no systematic changes (Fig. 5*F*; *P* = 0.038 and 0.941 for sensitivity and threshold; *n* = 790; sign tests).

Finally, as an additional test of our conclusion that the GCL is indeed conveying more information at high irradiance, we used the estimated LNP models to ask how accurately they could be used to reconstruct the original stimulus (note that this test was performed on a stimulus epoch not used for model identification; *Methods*). We found that neurons increasing their firing rate at higher light levels provided a more faithful representation of the original stimulus (Fig. 5 *G* and *H*; *P* ∼ 0; *n* = 385; sign test). This effect was not apparent in units whose firing rate either decreased or did not change (*P* = 0.053; *n* =107; sign test).

**Fig. 6.**
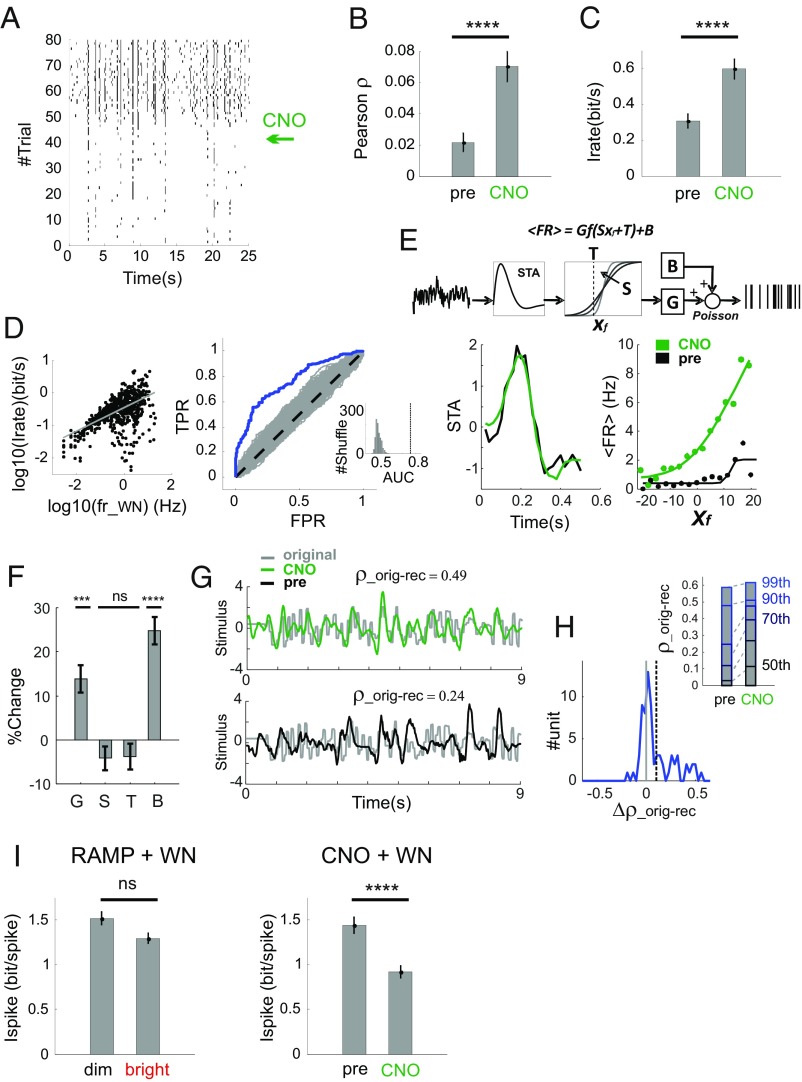
Impact of ipRGC-driven increases in firing on neural coding. (*A*) As in Fig. 5*A* for a representative CNO-responsive unit from an hM3Dq-expressing retina; arrow to *Right* shows time of CNO administration. (*B* and *C*) Trial-to-trial reproducibility (*B*) and information rate (*C*) for the block of 40 trials after (“CNO”) than before (“pre”) CNO delivery in units expressing increase in firing rates post-CNO (*P* = 0.00014, *P* ∼ 0 for G and B, *P* = 0.433 and 0.229 for S and T; *n* = 393; sign test). (*D*–*H*) As in Fig. 5 *D*–*H*, but applied to CNO+WN experiments in hM3Dq retinas; accordingly, distribution of Δρ_orig-red_ in *H* is for neurons exhibiting CNO-related increases in firing. (*I*) Coding efficiency, calculated as bits per spike, was equivalent in “dim” vs. “bright” portions of the irradiance ramp (*Left*) but significantly reduced following CNO delivery (*Right*). ***P < 0.001, ****P < 0.0001 for rank sum test.

The association between firing rate and information in the irradiance ramp data confirms that these parameters are closely linked but does not establish the nature of that relationship. One possibility is that they are unrelated responses to correlated changes in the visual stimulus. Our ramp-plus-white noise stimulus recapitulates the fundamental association between radiance mean and SD encountered in the real world (Fig. 1*B*). Thus, both mean radiance over time (equivalent to “irradiance”) and the amplitude of fast modulations in radiance (radiance SD) increase along the ramp. As a result, the changes in response gain and information rate could plausibly be responses to changes in the amplitude of the white-noise stimulus (radiance SD), with the increase in firing rate being an independent response to irradiance (radiance MEAN). Alternatively, there may be a causative relationship between firing and information rates/response gain, whereby an increase in irradiance drives higher firing rates that in turn allow for higher information rates/response gains. The ability of chemogenetic ipRGC activation to alter firing rate without changing the visual stimulus allows us to distinguish between these possibilities. To this end, we compared responses to the repeated white-noise stimulus in the epochs before and after CNO administration. We found that neurons whose firing rate rose after CNO administration in hM3Dq-expressing retinas also showed improvements in response reproducibility and information rate (Fig. 6 *A*–*C*; ρ: *P* ∼ 0, zval = 6.7, *n* = 122, rank sum test; info rate: *P* = 8 × 10^−5^, zval = 3.95, *n* = 122, rank sum test). Conversely, there were no significant changes in these properties in neurons whose firing rate did not increase (*SI Appendix*, Fig. S2*C*; ρ: *P* = 0.31, zval = 1, *n* = 271, rank sum test; info rate: *P* = 0.18, zval = 1.34, *n* = 271, rank sum test). Moreover, consistent with findings from the ramp experiments, we found that, at the single-unit level, firing and information rates were correlated in the CNO dataset (Fig. 6*D*, *Left*; Spearman’s ρ = 0.38, *P* ∼ 0), and that changes in information rates after CNO administration could be predicted from modulations in firing rate (Fig. 6*D*, *Right*; AUC = 0.76, *P* ∼ 0, shuffle test). As with the ramp experiments, not only baseline activity but also gain in visual responses increased when single units were fitted with an LNP model (Fig. 6 *E* and *F*; *P* = 0.00014, *P* ∼ 0 for gain and baseline; *P* = 0.433 and 0.229 for sensitivity and threshold; *n* = 393; sign test). Furthermore, stimulus reconstruction improved in units whose firing rate increased upon CNO application (Fig. 6 *G* and *H*; *n* = 71, sign test) but not in the remaining population (*P* = 0.0713; *n* = 123; sign test).

These results indicate that higher firing rates induced by irradiance ramps or CNO administration effectively enhance response gain and information transfer by RGCs. We next asked whether this had a cost in terms of coding efficiency. To explore this option, we computed a measure of coding efficiency (bits per spike) for GCL spiking under the white-noise stimulus across the irradiance ramp and before/after CNO activation. We found that coding efficiency was around 1.5 bits per spike at the start of the ramp and before CNO administration (Fig. 6*I*). This value is within the range reported in other systems and across species (1, 2, 25). In the case of the irradiance ramp, this figure was largely retained across changing ambient light even though both the amount of information and firing rate increased (Fig. 6*I*, *Left*; *P* = 0.63, zval = 0.47, *n* = 790, rank sum test). Conversely, while increases in firing caused by chemogenetic activation of ipRGCs were accompanied by an overall increase in information rate, the amount of information per spike decreased substantially (<1 bits per spike; Fig. 6*I*, *Right*; *P* < 2 × 10^−6^, zval = 4.75, *n* = 393, rank sum test).

## Discussion

Improvements in visual performance at higher ambient light are part of our everyday experience and well documented in the scientific literature. There are several established biological mechanisms that can contribute to this effect, including changes in pupil size and a raft of irradiance-dependent alterations in retinal circuitry that increase the spatiotemporal resolution of individual neurons. We report here an additional potential origin for irradiance-dependent increases in visual performance in the form of a progressive increase in RGC-maintained activity that has the effect of enhancing information flow at high irradiance.

The proposed function of irradiance-dependent control of RGC firing rate relies upon a causative relationship between this parameter and information flow. Our results indicate that this effect is mediated by ipRGC-dependent increases in gain of visual responses across a large RGC population. A close association between maintained activity and information rate has been previously described in neural systems across animal phyla (1–3, 25). Published descriptions of this phenomenon extend to reports of irradiance-dependent increases in neural activity and information content in elements of the early visual system in flies (26) and mammals (9, 27). However, it has been hard to separate cause and effect in such observations. Higher firing could be the driver allowing increased information transfer, or higher information flow could impose higher firing. Finally, the two events could be mechanistically unrelated, co-occurring thanks to the association between irradiance and the amplitude of fast changes in local light intensity in natural scenes (Fig. 1*B*) and in our experimental stimuli. The ability of chemogenetic activation of ipRGCs to increase firing without any change in the visual stimulus (Figs. 2 *C*–*H* and 3) has allowed us to distinguish between these various possibilities. Thus, the observation that increases in firing induced in this way are sufficient to enhance information, reveals a causative relationship between these parameters.

The finding that artificially increasing maintained activity can allow additional information to be transmitted by RGCs begs the question of why maintained activity is not simply higher under all conditions. Our data reveal that, although information rates can be increased simply by enhancing activity, this comes at the cost of a reduction in the amount of information per spike. Indeed, according to the definition used here (bits per spike), coding efficiency falls by ∼35% following chemogenetic activation of ipRGCs. In contrast, coding efficiency is held roughly constant at ∼1.5 bits per spike across similar alterations in maintained activity evoked by the irradiance ramp. Spike firing and associated synaptic activity can account for 80% of energy expenditure in the brain (28, 29), and task-dependent adjustments in neural activity (presumably to regulate energy usage) appear to be a conserved feature of nervous system design (30, 31). Viewed, in this context, the irradiance-dependent modulation of GCL activity appears to be a mechanism for trading off information content with energetic cost. At higher irradiances, more information can be transferred without impacting coding efficiency, while at lower irradiances the energetic cost of increased activity counteracts the benefit of higher rates of information.

The observation that activating ipRGCs to enhance GCL activity increases information implies that this manipulation recovers some bits of information that are otherwise lost. This raises the question of at what point(s) in the retinal circuitry information loss is ameliorated. Melanopsin could increase response gain of those ganglion cells (including ON alphas) in which it is expressed (32). However, the impact of irradiance and ipRGC activation extends across the ganglion cell population, including cells with OFF response polarity. The simplest possibility is that it is the activity of these RGCs themselves that is ordinarily limiting, and that ipRGCs ameliorate information loss at this final stage of retinal processing at higher irradiance. However, ipRGCs have connectivity to allow them to influence the visual signal at all steps of its transition through the retina. They make gap junction connections with other cells in the GCL (10, 12), send axon collaterals into the inner plexiform layer (13), target dopaminergic amacrine cells (11), and can influence the activity of bipolar cells (33) and even photoreceptors (17). They may thus be able to enhance nonspiking activity in the outer retina to limit visual information loss in photoreceptors, horizontal or bipolar cells (34). Nonspiking activity is also energetically expensive, and in fly photoreceptors has been linked to irradiance and information content (26). Thus, the energy constraints relevant for spiking activity in RGCs could also apply to other retinal cell types.

A related question is whether irradiance-dependent changes in firing (and information) are restricted to particular subtypes of RGC. In theory, active control over firing could provide a mechanism of biasing retinal output toward types of information most relevant under different lighting conditions. While our study does not include an exhaustive test of this hypothesis, our data rather support the alternative view that the irradiance response is largely unselective. Increase in firing across the irradiance ramp showed a monomodal distribution and responses to the ramp and CNO appeared across ON and OFF ganglion cells.

While much of the analysis presented here concerns the consequences of changes in maintained activity for the transfer of higher-frequency visual signals, it is certainly possible that irradiance-driven changes in firing rate are themselves also a method of conveying information. There is a growing body of evidence that neurons can convey multiple types of information by multiplexing across different timescales (6, 35). In this context, the same RGCs could convey information about visual patterns in the fine timing of spikes, and irradiance in their maintained activity. The latter irradiance code appears in the thalamus (9) and could be exploited at higher levels to support perception of ambient light (36) or as contextual information for visual prediction and for associative learning (37).

The description of active control over firing rate as a mechanism for adjusting information capacity in the optic nerve raises the question of whether similar phenomena await description in other sensory systems. The critical feature of the irradiance response described here is that a measurable independent variable (irradiance) predicts the amount of sensory information available to encode. It will be interesting in the future to determine whether similar adjustments in tonic activity are associated with other physical parameters with predictive value for information content (e.g., time of day) in vision or other sensory systems.

## Methods

### Animals.

Experiments were in accordance with the UK Animals (Scientific Procedures) Act 1986 approved by Home Office (PPL Number 70/8918) and performed on adult (3–6 mo) *Opn4*^*Cre/+*^*;Z/EGFP* mice housed in a 12-h dark/light cycle at 22 °C with food and water available ad libitum.

### Intravitreal Injections.

A volume of 2.5 μL of AAV2-hSyn-DIO-hM3D(Gq)-mCherry vector (2.313 × 10^13^ genomic particles per mL; The University of North Carolina Vector Core) or, for controls, virus-free PBS in combination with hyaluronan lyase and heparinase III (0.2 U each) was introduced gradually to the vitreal space of anesthetized *Opn4*^*Cre/+*^ mice over 1 min. Mice were allowed at least 6 wk to recover before being used in experiments. For c-Fos studies, CNO (ab141704; Abcam) was administered via i.p. route (5 mg/kg), while for in vitro electrophysiology 5 µM CNO was added to aCSF.

### Electrophysiology.

Mice were held in the dark for 12 h (to enhance integrity of dissected retina) before cervical dislocation under dim red light (50 nW⋅cm^−2^, λ > 650 nm). Still working under dim red light, retinas were isolated in carboxygenated (95% CO_2_, 5% CO_2_) aCSF, with concentrations (in mM): 118 NaCl, 25 NaHCO_3_, 1 NaH_2_PO_4_, 3 KCl, 1 MgCl_2_, 2 CaCl_2_, 10 C_6_H_12_O_6_, and 0.5 l-glutamine (Sigma-Aldrich). The retina was incised radially multiple times along the edge to maximize planarization and then mounted onto a 256-channel MEA (256MEA200/30iR-ITO; Multichannel Systems) with the GCL facing down onto the electrodes. A Cyclopore membrane (5-μm pores; Whatman) held the retina in place while being weighed down by two stainless-steel anchors (∼0.75 g each) bearing a framework of parallel polyimide-coated fused silica capillaries (TSP320450; Polymicro Technologies).

Electrophysiological signals were filtered (200-Hz high pass, Butterworth second order) and recorded in the form of spikes at a sampling rate of 25 kHz using MC_Rack software (Multi Channel Systems) through a USB-MEA256 amplifier. The retinal explant was superfused with carboxygenated aCSF at a rate of 3.5 mL/min using a peristaltic pump (120U; Watson Marlow) and maintained at 32 °C using a TC01 controller (Multi Channel Systems) regulating the temperature of a copper plate below the MEA. Stimulation files were merged offline in MC-DataTool (Multichannel Systems) and sorted in Offline Sorter (Plexon). Spike times were exported to Matlab (The Mathworks) for analysis via NeuroExplorer (Plexon).

#### Visual stimuli.

Ex vivo retinas were illuminated using a combination of violet, blue, cyan, and yellow (λ_max_ at 400, 430, 480, and 560 nm, respectively) elements of a multispectral LED light source (SPECTRA X light engine; Lumencor). The intensity distribution of the temporal white-noise modulations were as described previously (9) and produced by pulse width modulation of LEDs controlled via the counter channels of a NI USB-6343 card and LabVIEW software (National Instruments Corporation). The irradiance ramp was produced by a gradient neutral-density filter wheel (Newport Corporation) controlled by a stepper motor (Newport Corporation) to provide 5.12 log-equally spaced steps per second, each step corresponding to 0.2% increase in irradiance. The filtered light was then collected by a liquid light guide and delivered to the mouse retina mounted on the MEA. The effective irradiance for each photopigment was calculated as previously (33).

#### Field recordings.

The radiance measurements shown in Fig. 1 *A* and *B* were obtained by using a custom-built portable device. Raw readings were obtained with a linear photodiode (Thorlabs). Data were sampled at 5 Hz, and acquisition was controlled by an Arduino UNO board (Arduino LLC). Radiance measurements were then calibrated by using a LIFX Color 1000 bulb.

### c-Fos Immunohistochemistry.

CNO (5 mg/kg) was administered i.p. to hM3Dq-expressing and control *Opn4*^*Cre/+*^ mice under dim, deep red light (50 nW⋅cm^−2^; λ > 650 nm). After 90 min in the dark, mice were deeply anesthetized with i.p. injection of urethane [2.2 g/kg; 30% (wt/vol); Sigma-Aldrich] and perfused transcardially with 0.9% saline followed by 4% paraformaldehyde. Brains and eyes were removed, postfixed overnight in 4% paraformaldehyde, and then transferred to 30% sucrose for cryoprotection for 2 d.

Coronal brain sections (50 μm) were stored free floating in 0.1 M phosphate buffer. Sections were incubated in 2% hydrogen peroxidase for 20 min and then in blocking buffer (0.1 M phosphate buffer, 3% Triton X-100, and 0.5% normal goat serum) for 1 h. Sections were incubated in rabbit anti–c-Fos (Calbiochem; Ab-5; 1:20,000) overnight at 4 °C, and then visualized with a goat anti-rabbit Vectastain horseradish peroxidase kit (Vector Labs) and DAB (Vector Labs) as a chromogen. Sections were mounted on microscope slides and coverslipped with DPX. Images were acquired using the 3D Histech Pannoramic 250 Flash II slide scanner with 20×/0.80 Plan Apo objective. A blinded scorer applied 3D Histech Pannoramic Viewer and ImageJ to count c-Fos–positive cells and measure the area of region counted from. Between three to five sections covering the same portion of the dLGN (according to coordinates in ref. 38) in each animal were analyzed. The number of c-Fos–positive cells was normalized to the area of the region and compared between hM3Dq-expressing and control animals using a two-tailed unpaired *t* test (GraphPad Prism 6.04).

Immunohistochemistry on retinal sections was performed as previously described (16). hM3Dq-mCherry expression was revealed by a rabbit anti-dsRed primary (Clontech; 632496; 1:1,000) and Alexa 546-conjugated donkey anti-rabbit (1:300; Life Technologies), and GFP with chicken anti-GFP (Abcam; ab13970; 1:1,000) and GFP-expressing ipRGCs were revealed by Alexa 488-conjugated donkey anti-chicken (1:300; Life Technologies). c-Fos staining employed rabbit anti–c-Fos primary (Cell Signaling; 9F6; 1:250) and Alexa 488-conjugated goat anti-rabbit secondary (1:300; Life Technologies) antibodies. RBPMS staining employed guinea pig anti-RBPMS primary (1:300; PhosphoSolutions), and Alexa 650-conjugated donkey anti-guinea pig (1:300; Life Technologies) antibodies. Images were collected on either a Leica TCS SP5 AOBS inverted confocal using a 40×/0.50 Plan Fluotar objective and 1.5× confocal zoom or on an Olympus BX51 upright microscope using a (20×/0.5 UPlan FLN) objective and captured using a [Coolsnap ES2 camera (Photometrics)] through Metavue, version 7.8.4.0, software (Molecular Devices). The number of c-Fos–positive cells was normalized to the length of a retinal section. These data were analyzed using a two-tailed unpaired *t* test (GraphPad Prism 6.04).

### Data Analysis.

#### Evaluation of ramp responses.

Firing rate was estimated along the ramp by using time bins corresponding to half log unit steps in irradiance. To measure the relative increase in firing rate, we calculated the logarithmic ratio between the lowest and highest irradiance levels [log_10_(fr_ratio_)] on the *y* axes of Fig. 2 *F* and *G*). As additional measure of irradiance sensitivity, we used the *R*^2^ values obtained by fitting firing rate responses as a log linear function of irradiance (*R*^2^ on the *x* axes of Fig. 2 *F* and *G*).

#### Estimation of CNO effects on firing rate.

The average firing rate of each unit was estimated in of the 20 WN epochs before and after CNO delivery. We then tested each unit for the possibility that CNO induces a systematic change in average firing by performing an unpaired rank sum between firing-rate distributions measured before and after CNO delivery.

#### ON–OFF classification.

STAs were calculated for each isolated unit. For the ramp protocol, we calculated six STAs per unit, each corresponding to a half log unit increment in irradiance. For the WN+CNO experiments, the STAs were separately calculated before and after CNO delivery. Each STA was tested (*t* test; α = 0.01) for significance by comparing its energy with a null distribution obtained by randomly shuffling the times of spike occurrence along the time of the recording (20 repeats). All significant STAs (*n* = 6,983) were standardized and projected along the first five principal components (the number of components was determined as the smallest set that could explain >75% of the STA variance). We then applied a kmeans clustering for two groups to separate units into ON and OFF polarity (Fig. 2*D*).

### Information Calculations.

#### Definition of mutual information.

In this study, we used fast pseudorandom changes in illumination that are known to elicit precisely timed firing patterns in RGCs. A main aim of the study is to compare how coding of these patterns changes at the level of individual units when those units are biased toward higher levels of basal firing activity. According to our experimental design, the same relative changes in illumination occur at the same time in each condition and individual neurons express similar firing patterns, but the magnitude of basal activity and evoked responses changes according to our manipulations (Fig. 5 *A* and *E*). As these manipulations typically resulted in higher firing, we asked whether and to what extent these “extra spikes” enhance or degrade neural coding. To answer this question, we used Shannon’s mutual information defined as follows:

I(S;R)=H(R)−H(R|S),

where *H*(*R*) and *H*(*R|S*) represent the response entropy and the noise entropy, respectively. The two entropy terms are defined as follows:

$$H\left(R\right)=-\sum_{r}P\left(r\right)\log_{2}P\left(r\right),$$

$$H\left(R|S\right)=\;-\frac{1}{N}\sum_{t}\sum_{r}P\left(r\text{|}t\right)\log_{2}P\left(r\text{|}t\right),$$

where ***r*** represents the neural response and ***N*** represents the number of time windows in each trial. The response ***r*** was calculated in a time window of duration *T* as the *L*-dimensional vector of firing rates ***r*** *=* [*r*_**[0,**__**∆*t*)**_, *r*_[∆*t*,2*∆*t*)_**, …,** *r*_**[(**l__−__1)*∆*t*,__L*∆*t*)_] within *L* adjacent subwindows of duration **∆*t*** such that T = L*∆*t*. The response entropy is determined by the time-averaged distribution of firing rates *P*(***r***) = <*P*(***r****|t*)>_*t*_, where *P*(***r****|t*) is the probability of response ***r*** in the time window [*t* − *T*/2, *t* + *T*/2) and <.>_*t*_ denotes averaging across time windows. For a neuron that is insensitive to changes in illumination, then *P*(***r****|t*) = *P*(***r***) at any given time ***t*** and the mutual information will be zero.

#### Identification of the coding timescale.

Given a constant and relatively large window *T*, both *P*(***r***) and *P*(***r****|t*) are estimated on subwindows of finite duration **∆*t*** and the choice of **∆*t*** will affect the estimate of mutual information. Although ideally we would like **∆*t*** to be as small as possible, this would result in a very high-dimensional response vectors that cannot be properly sampled with a finite number of stimulus trials (see *Bias correction*). On the other hand, an extremely conservative choice of **∆*t*** will fail to capture the temporal precision of the neural responses. To identify a suitable trade-off, we first choose a value of *T* = 200 ms because comparable with the latency of the STAs peaks (Fig. 1*G*). We then calculated the mutual information by changing the number of subwindows *L*. As mutual information tended to saturate with *L* (*SI Appendix*, Fig. S2*D*), we selected **∆*t*** = 50 ms (corresponding to *L* = 4), which provided a good estimate of the temporal precision for neural responses in our data.

#### Information rate.

Information rate is defined as $\underset{T\to \infty }{\lim}I\left(S;R\right)/T$. In practice, we estimated the information rate, in bits per second, as the slope of *I*(*S*;*R*) as function of *T* (*T* = 50, 100, 150, and 200 ms), given a fixed temporal subwindow (**∆*t*** = 50 ms).

#### Coding efficiency.

To evaluate coding efficiency, we estimated the information per spike in bits per spike (for *T* = 200 ms, **∆*t*** = 50 ms) as *I*_*spk*_ *= I*(*S*; *R*)/(T*<firing rate*>*), where <.> denotes averaging across trials and time windows.

#### Bias correction.

Given a finite number of trials, the naive calculation of the response and noise entropies returns negatively biased values. As the undersampling is more serious for the noise entropy, the naive estimate of mutual information is positively biased. To correct the mutual information estimates, we used the information decomposition as described in ref. 39:

$$I_{Sh}\left(S;R\right)=H\left(R\right)-H\left(R\text{|}S\right)+H_{sh}\left(R\text{|}S\right)-H_{ind}\left(R\text{|}S\right),$$

where *H*_*ind*_(*R|S*) is calculated by ignoring temporal correlation between subwindows, that is, by using the conditional distributions $P_{ind}\left(r\text{|}t\right)=\prod_{n=1}^{L}P\left(r_{\left[(n-1)*\Delta t,n*\Delta t\right)}|t\right)$, while *H*_*sh*_(*R|S*) is estimated as *H*(*R|S*) but after shuffling the components of the response vector **r** across trials. In the theoretical limit of an infinite number of trials, the last two terms will cancel out. In practice, the entropy term *H*_*ind*_(*R|S*) is better sampled than *H*_*sh*_(*R|S*) and the difference *H*_*sh*_(*R|S*) − *H*_*ind*_(*R|S*) provides an effective correction for the positive bias. For the final estimate of *I*_*sh*_(*S*;*R*), the value of each entropy term was then extrapolated in the limit of an infinite number of trials by using the quadratic extrapolation described in ref. 40.

#### Control for finite duration stimulus.

Given the finite duration of our WN sequence, the space of all possible stimuli cannot be explored during the experiment. Therefore, it is possible that the particular pseudorandom sequence of our illumination values provides a bias itself in the estimate of the amount of information conveyed by individual units. To control for this possibility in CNO experiments, we divided each trial in two separated epochs of temporal white noise. We then separately calculated mutual information for each epoch. The results for each unit were well matched (*SI Appendix*, Fig. S2*E*), indicating that the duration of each stimulation epoch is enough to capture its coding properties.

#### Predicting changes in mutual information from changes in firing rates.

We wanted to determine whether and to what extent the level of firing rates were predictive of the increments/decrements in information conveyed about changes in illumination during full-field noise stimulation. For each unit, we used relative changes in firing rate *X =* (*fr*_1_ − *fr*_0_)/(*fr*_1_ + *fr*_0_) as continuous independent variable and *Y* = *Heaviside*(∆*I*) (0 for ∆*I* < 0, 1 for ∆*I* ≥ 0) as binary dependent variable. We then fitted a logistic model,

$$P\left(Y\text{|}X\right)=\frac{1}{1+e^{-\left(\beta 0+\beta 1*X\right)}},$$

and used it as binary classifier. By systematically changing the threshold of the classifier, we estimated the AUC from the receiver operating characteristic curve. Values of AUC larger than 0.5 indicate that changes in firing rate are predictive values of the changes in the amount of information conveyed. To assess statistical significance, we performed a shuffle test (1,000 shuffle; *Insets* in Fig. 5 *D* and *H*, *Right*).

#### LNP identification.

To better understand the effect of ipRGC excitation on processing of individual neurons, we fitted the input–output transformation by using a parameterized LNP model (24). We used the STA to filter the stimulus (*x*_*f*_). Then we fitted the relation between *x*_*f*_ and the unit’s firing rate by using the following parameterized function:

$$FR\left(x_{f}\right)=Gf\left(Sx_{f}+T\right)+B,$$

where the *f*(***·***) is the cumulative normal density and the parameters have the following interpretation: *G*, response gain; *S*, sensitivity to nonlinearity; *T*, threshold; and *B*, baseline activity.

This approach is similar to that described by Chichilnisky (24), the only difference being in the additional parameter “*B*” that we used to account for stimulus-unrelated baseline activity.

#### Stimulus reconstruction.

To further evaluate the ability of individual neurons to represent an incoming stimulus, in addition to the previously described measure of mutual information, we used a decoding approach. We divided the stimulation sequence into two epochs that we used respectively for estimating the LNP model (training set) and perform maximum a posteriori decoding (test set). Decoding performances on the test set were then evaluated by measuring Pearson’s linear correlation between the original and the reconstructed stimulus (ρ_orig-rec_ in Figs. 5 *G* and *H* and 6 *G* and *H*).

## Supplementary Material

Supplementary File
