# Supplementary material for: Photoreceptive retinal ganglion cells control the information rate of the optic nerve
Source: Proc Natl Acad Sci U S A. 2018 Nov 28;115(50):E11817–26. doi: 10.1073/pnas.1810701115 (PMC6294960; doi:10.1073/pnas.1810701115)
Supplement: Supplementary File [file pnas.1810701115.sapp.pdf]

Supplementary Figures:

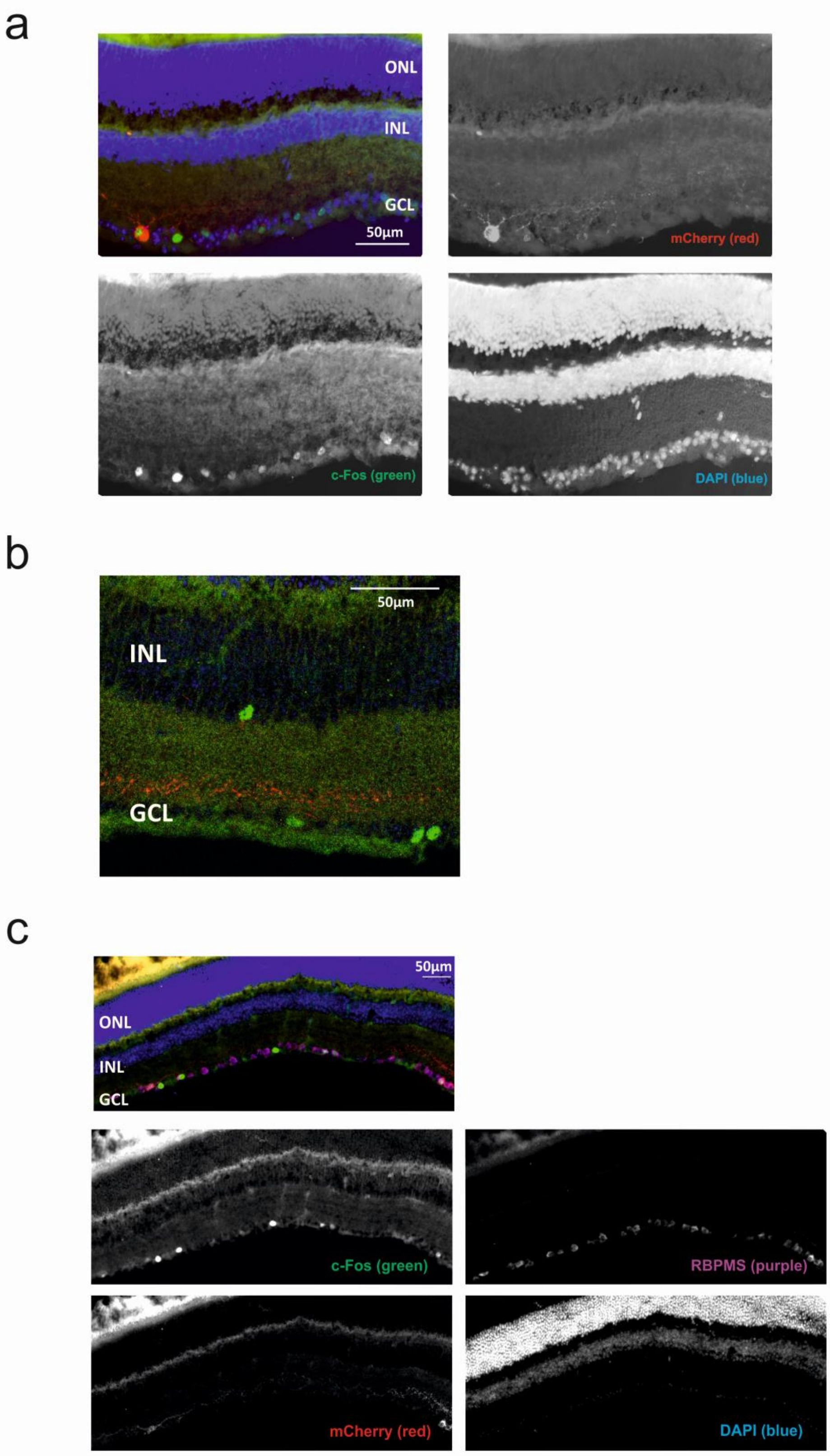

www.pnas.org/cgi/doi/10.1073/pnas.1810701115

Figure S1. c-Fos induction in retinas from hM3Dq-expressing mice. **(a)** Representative retinal section following immunohistochemistry for c-Fos and mCherry and counterstained with DAPI. Top left panel shows all signals superimposed, with c-Fos and mCherry immunoreactivity in green and red respectively, and DAPI in blue. Monochrome images show signal isolated signals for mCherry, DAPI and c-Fos (clockwise from top right). **(b)** c-Fos expression in INL (green arrow) and colocalized with mCherry in GCL (red arrow). **(c)** A representative retinal section following immunohistochemical staining for c-Fos, mCherry and RBPMS. Top left shows composite image with immunoreactivity for c-Fos, mCherry and RBPMS shown respectively as green, red and purple and DAPI nuclear counterstain (blue). Individual channels shown in monochrome images for c-Fos, RBPMS, DAPI and mCherry (clockwise from top left). Scale bar, 50 μm.

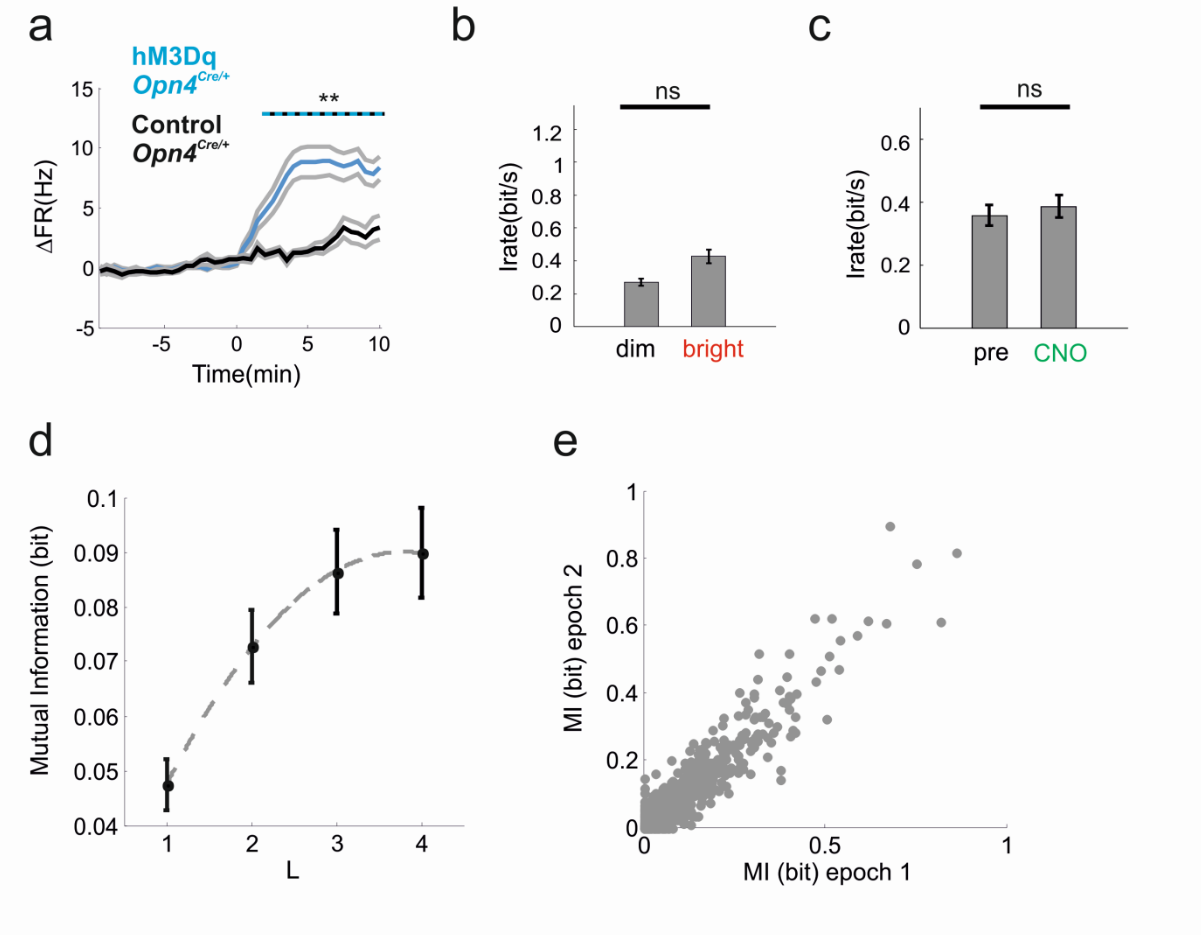

Figure S2. (a) As **Fig. 4b** but restricted to the 20% units in hM3Dq and controls with highest increase in activity following CNO. Note that while hM3Dq units express a sharp increment after CNO control units express a slow drifting behaviour. **(b)** Information rate for units that did not express increase in firing rates across the ramp (p = 0.27, zval = 1.09, n = 326, ranksum test). **(c)** Information rate for units that did not express increase in firing rates after CNO delivery (p = 0.18, zval = 1.34, n = 271, ranksum test). **(d)** Mutual information calculated for time window T (T = 200ms) as function of different number of sub-windows L. **(e)** Mutual information (T = 200ms, L = 4) calculated for each unit during CNO experiment during the 1st and 2nd epoch of temporal white noise stimulation (respectively x & y axes). Values obtained for the 2 epochs are well matched (Pearson's correlation coefficient = 0.947) indicating the stimulation sequences are long enough to capture coding properties of individual units.
